# Continuity of primary care for type 2 diabetes and hypertension and its association with health outcomes and disease control: insights from Central Vietnam

**DOI:** 10.1186/s12889-023-17522-6

**Published:** 2024-01-02

**Authors:** Quynh-Anh Le Ho Thi, Peter Pype, Johan Wens, Huy Nguyen Vu Quoc, Anselme Derese, Wim Peersman, Nhon Bui, Huyen Nguyen Thi Thanh, Tam Nguyen Minh

**Affiliations:** 1 Family Medicine Center, Hue University of Medicine and Pharmacy, Hue University, Hue, Vietnam; 2 Department of Public Health and Primary Care, Faculty of Medicine and Health Sciences, Ghent University, Ghent, Belgium; 3 Department of Family Medicine and Population Health, University of Antwerp, Antwerp, Belgium; 4 Department of Obstetrics and Gynecology, Hue University of Medicine and Pharmacy, Hue University, Hue, Vietnam; 5 Research Group Social and Community Work, Odisee University of Applied Sciences, Brussels, Belgium; 6 Department of Rehabilitation Sciences, Ghent University, Ghent, Belgium; 7 Phu Vang District health center, Thua Thien Hue province, Hue, Vietnam

**Keywords:** Continuity of care, Hypertension, Type 2 diabetes mellitus, Disease control, Primary care, Vietnam

## Abstract

**Background:**

Vietnam is undergoing a rapid epidemiological transition with a considerable burden of non-communicable diseases (NCDs), especially hypertension and diabetes (T2DM). Continuity of care (COC) is widely acknowledged as a benchmark for an efficient health system. This study aimed to determine the COC level for hypertension and T2DM within and across care levels and to investigate its associations with health outcomes and disease control.

**Methods:**

A cross-sectional study was conducted on 602 people with T2DM and/or hypertension managed in primary care settings. We utilized both the Nijmegen continuity of care questionnaire (NCQ) and the Bice - Boxerman continuity of care index (COCI) to comprehensively measure three domains of COC: interpersonal, informational, and management continuity. ANOVA, paired-sample t-test, and bivariate and multivariable logistic regression analysis were performed to examine the predictors of COC.

**Results:**

Mean values of COC indices were: NCQ: 3.59 and COCI: 0.77. The proportion of people with low NCQ levels was 68.8%, and that with low COCI levels was 47.3%. Primary care offered higher informational continuity than specialists (*p* < 0.01); management continuity was higher within the primary care team than between primary and specialist care (*p* < 0.001). Gender, living areas, hospital admission and emergency department encounters, frequency of health visits, disease duration, blood pressure and blood glucose levels, and disease control were demonstrated to be statistically associated with higher levels of COC.

**Conclusions:**

Continuity of primary care is not sufficiently achieved for hypertension and diabetes mellitus in Vietnam. Strengthening robust primary care services, improving the collaboration between healthcare providers through multidisciplinary team-based care and integrated care approach, and promoting patient education programs and shared decision-making interventions are priorities to improve COC for chronic care.

**Supplementary Information:**

The online version contains supplementary material available at 10.1186/s12889-023-17522-6.

## Introduction

The prevalence of NCDs is predicted to increase rapidly over the next decade [[1]]. Like other low and middle-income countries (LMICs), Vietnam is facing the challenge of poor control of NCDs, especially hypertension and type 2 diabetes mellitus (T2DM) [2]. More than 70% of people diagnosed with hypertension/diabetes had not achieved control of their diseases [3, 4]. These issues pose a tremendous burden on already weakened health systems in LMICs, including Vietnam [5].

Vietnamese healthcare delivery is decentralized into four levels: commune, district, provincial, and central. Despite efforts to better respond to the healthcare needs of hypertension and T2DM through several national strategies [6], the delivery of NCD services is still hospital-and-specialist-centric, particularly for T2DM. A previous study showed that only 53% of commune health centers (CHCs) offered diabetes services, with only 3% having at least one type of diabetes medication (metformin, glibenclamide, or insulin), while 64% of CHCs offered treatment services for cardiovascular diseases [7]. Bypassing primary care and overload of the upper-level health facilities, lack of investment of medication and equipment for NCDs at primary care, lack of intersectoral coordination and direction for NCDs management as well as evidence-based research have led to an inefficient and fragmented health system for NCDs services [7, 8]. Moreover, since 2016, national law on health insurance schemes allowed insured individuals to seek care at any CHC or district-level health facility (DHC) within the same province, instead of being restricted to their registered facility [9]. This law has improved the accessibility to quality healthcare services but has also led to a lack of follow-up and continuous care from a specific physician for people with NCDs.

Continuity of care (COC) is widely acknowledged as a benchmark for high-quality care services and an efficient healthcare system [10]. Remarkably, the COVID-19 crisis revealed a weak COC system and disrupted care for people with NCDs who need long-term care [11]. Initially introduced in the 1950s, the concept of COC referred to care provided by the same health professional (interpersonal continuity) [12]. The World Health Organization (WHO) defined COC as the degree to which a series of discrete healthcare events is experienced by people as coherent and interconnected over time and consistent with their health needs and preferences [13]. A multidisciplinary review summarized three major common themes within the different concepts of COC: (1) Interpersonal continuity - personal relationship between patient and care provider, (2) Informational continuity - communication of relevant patient information between providers, and (3) Management continuity - cooperation between providers [10, 12, 14]. With the multi-dimensional construct of the COC concept, there is a broad spectrum for measuring COC in different aspects due to the differences in the healthcare system, medical conditions, and resource availability [15]. Several reliable and valid instruments to measure COC were developed and available. However, using multiple tools is recommended to limit the disadvantages of a lack of perfect measurement tools and to ensure a comprehensive assessment of different aspects of the care continuum [16, 17].

The advantages of COC have been documented in many countries [16–25]. Previous studies demonstrated that a high level of continuity of care contributed to reducing mortality [18], hospital and emergency admission, and healthcare costs [19–21], improving treatment adherence and disease control [22–24] as well as leveraging patient satisfaction and quality of life [25]. Chronic care requires coordinated care among the multidisciplinary team and multi-care levels to ensure patients do not feel frustrated when they visit different healthcare providers in the referral process and do not receive *inconsistent* advice and information from various providers [26]. Studies on COC in primary care settings for NCDs in Vietnam have been limited to date. Given the healthcare system’s focus on hospitals and secondary care for NCDs, a robust and comprehensive approach to evaluating COC is strongly needed. This study aimed to determine the extent of COC for hypertension and T2DM within and across care levels and to investigate its associations with patients’ health outcomes and disease control.

## Materials and methods

### Study design and setting

This descriptive cross-sectional study was conducted among people with hypertension and/or T2DM from May 2019 to February 2020. The study took place in Thua Thien Hue province, located in the Central region of Vietnam. All study methods adhered to ethical guidelines and regulations approved by the Ethical Committee in Biomedical Research of Hue University of Medicine and Pharmacy. Data collection was completed before the COVID-19 pandemic emerged in Vietnam. Notably, Thua Thien Hue, our study area, remained COVID-19-free throughout 2020, ensuring the pandemic had no impact on our participant selection, data collection, and interviews. Hypertension management services are more readily available than diabetes management services in CHCs in Vietnam. In Thua Thien Hue province, 97.4% of CHCs offer management and treatment services for hypertension, whereas the availability for diabetes is notably lower at 40.8% [27, 28].

### Study population

The study aimed to include approximately 682 people based on the ratio method of estimation, which assumed a 69.7% proportion of people achieving a high level of COC [29], a 95% confidence interval (CI), marginal error (d) of 5%, a design effect of 2, and a 5% non-response rate. Eligible participants included those who (1) had been diagnosed with T2DM and/or hypertension, (2) were managed at out-patient clinics at primary care levels (CHCs and DHCs) in Thua Thien Hue province, and (3) had at least two visits for care providers during the last 12 months before the interview. The study employed a multi-stage sampling technique in Thua Thien Hue province, which comprises nine districts: two mountainous, three urban, and four rural. Firstly, one district from each category was randomly selected via a random drawing method. We employed simple random sampling within each chosen district to select four communes. Lastly, we reviewed electronic medical records and chronic disease management booklets from 12 selected CHCs to define individuals managed at the primary care level. Specifically, we sought individuals with diabetes and/or hypertension who had records of medical care and documented follow-up care in these sources using systematic random sampling. Eligible participants meeting the inclusion criteria were invited to participate, with written informed consent obtained from all before their involvement.

### Study instrument

The respondents were invited to visit the CHC for a structured face-to-face interview. The questionnaire comprised four parts: (1) socio-demographic and health insurance information, (2) participants’ clinical profile characteristics and health-related quality of life, (3) health care services utilization in the previous 12 months, (4) participants’ experience of COC across care levels (primary and specialist care).

Participants were asked to present their health insurance cards, medical booklets, and poverty certificates if they self-identified as poor. Poverty was defined as household income below 1,000,000 VND (approximately 40 EUR) per person monthly in rural areas and 1,300,000 VND (approximately 51 EUR) per person monthly in urban areas [30]. We assessed comorbidities using the Charlson Comorbidity Index (CCI), categorizing patients as mild (CCI scores 1–2), moderate (CCI scores 3–4), and severe (CCI scores ≥5) [31]. Health-related quality of life (QoL) was measured with the EuroQOL-5 Dimensions-5 Levels (EQ-5D-5L), which has a score range of − 0.5115 to 1 for the Vietnamese population [32]. The EQ-5D-5L index of the general Vietnamese population of 0.91, found in a previous study in Vietnam, was used as the cutoff point to classify participants into two groups of low (< 0.91) or high QoL ((≥ 0.91) [33]. Weight, height, waist-hip circumferences, blood pressure, and fasting blood glucose test were measured during the interview. Blood pressure (BP) was measured on two occasions, separated by a short break. According to the guideline of the Vietnam Ministry of Health [34], the average of two readings of blood pressure was used and categorized into three groups: normal BP (< 130/85 mmHg), high-normal BP (systolic BP 130–139 mmHg and/or diastolic BP 85-89 mmHg), and high blood pressure (systolic BP ≥ 140 mmHg and/or diastolic BP ≥ 90 mmHg). Participants were asked not to eat or drink anything from 10 to 12 hours before the fasting blood glucose test, which a nurse performed. The fasting blood glucose results were classified into three groups: normal (< 5.6 mmol/l; 100 mg/dL), impaired glucose tolerance (5.6–6.9 mmol/l), and high blood glucose (≥7 mmol/l; 126 mg/dL) [34].

**Fig. 1:**
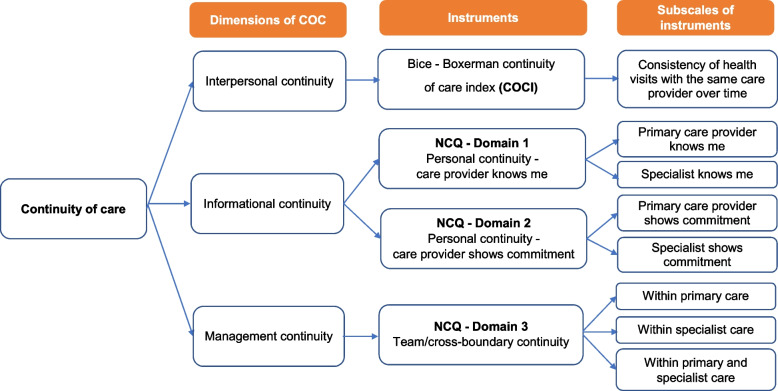
Conceptual framework of COC measurement in this study

For a comprehensive assessment of COC among the study population, we designed our study within a multi-dimensional framework of COC by using two distinct instruments: the Bice - Boxerman continuity of care index (COCI) [35] and the Nijmegen continuity of care questionnaire (NCQ) [36] (Fig. 1). The COCI primarily evaluates healthcare visit concentration from the provider side and suits large-scale statistical analysis and medical service fragmentation assessment [15–17]. In contrast, the NCQ focuses on the qualitative aspects of COC, including patient-provider relationships and experiential care quality.

We carefully considered the implications of using different instruments and subscales for each COC dimension through the definition and multi-dimensional frameworks of COC. Our primary objective is to comprehensively understand COC within our specific context. In Vietnam, the overlapping scope of practice between DHCs and CHCs in hypertension and diabetes care highlights the need to measure COC across the entire primary health system. Using NCQ enriched our understanding of the COC within primary care and across primary and secondary care settings. Our approach allows us to capture both structural/provider-oriented and patient-oriented aspects of COC, providing a more holistic assessment.

The COCI was calculated using the following formulas wherein M, n_j_, and N denote the total number of physicians, the number of visits to a physician j, and the total number of physician visits, respectively [35]:

$$\textrm{COCI}=\frac{\sum \limits_{j=1}^M{n}_j^2-N}{N\left(N=1\right)},$$

The COCI score values between 0 and 1. The closer the COCI score was to 1, the higher relational continuity of care was obtained. The COCI was transformed into two groups, low- and high-continuity groups. High continuity was defined as an index value of 0.75 or over, and low continuity as less than 0.75, as in previous studies in Korea (2019) [29] and Canada (2009) [37].

The NCQ was developed by Uijen AA et al. (2012) [36], validated in different countries [24, 38–40] and mainly applied in the out-patient, primary and secondary care settings. The Vietnamese version of this instrument was validated by forward and backward translating and testing to verify if the translation was correct and appropriate to the Vietnamese context. The NCQ instrument comprises 28 items of three COC domains: (1) personal continuity - care provider knows me; (2) personal continuity - care provider shows commitment; and (3) team/cross-boundary continuity. Each domain includes two subscales: the first assesses COC experience in primary care settings, and the second assesses COC experience in specialist care. For the team/cross-boundary continuity domain, besides considering collaboration between primary care providers and collaboration between specialists, an additional subscale of collaboration between primary care and specialist care is also included [36]. This NCQ scale uses a five-point Likert scale for scoring: 1 (strongly disagree), 2 (disagree), 3 (neutral), 4 (agree) to 5 (strongly agree). Each subscale eventually has a mean score. NCQ scores below four are interpreted as low and mean scores of four or higher as high continuity of care [41].

### Statistical analysis

Epidata 3.1, SPSS 18.0, and MS. Excell were utilized for data entry and analysis. Chi-square analysis was utilized to assess the association between COCI and NCQ scores and the demographic characteristics of participants. Paired sample T-test was performed to identify the difference in COC provided by primary care and hospital/specialist care. The one-way analysis of variance (ANOVA) and posthoc tests were also used to measure the pair-wise differences in COC between groups of hypertensive patients, diabetic patients, and those with both diseases. We employed the Pearson correlation coefficient to explore the correlation between COCI and each of the seven subscales of the NCQ as well as between the subscales of NCQ. Simultaneously, bivariable and multivariable logistic regression analyses were performed to examine the predictors of COC among people with hypertension and/or T2DM. Variables with a *p*-value < 0.2 at bivariable logistic regression analysis were entered into the multivariable logistic regression model. A p-value less than 0.05 was considered statistically significant.

## Results

A total of 602 respondents were involved, resulting in an 88.3% response rate. Table 1 provides an overview of study population, indicating that 56.3% of participants were female. The average age of participants was 64.9 (SD = 12.1) years old. The majority of respondents were living in remote areas (71.1%), had personal health insurance (99.7%), and registered their first point of care at CHCs (93.2%). 42.2% of participants had a high rate of health-related quality of life. The average number of co-morbidities among participants was 1.4 (SD 0.8); the sample comprised a high proportion of mild CCI (88.7%). Nearly 70% of participants lived with NCDs for 5 years or less and were poorly controlled with their NCD conditions.

**Table 1:** Demographic and clinical characteristics of respondents (*n* = 602)

Characteristics, n (%)	Frequency (n)	Percent (%)
**Gender: Female**	339	56.3
**Age**		
< 45	32	5.3
45–64	258	42.9
65–74	173	28.7
≥ 75	139	23.1
**Area**		
Urban	174	28.9
Remote^a^	428	71.1
**Highest qualification**		
Primary school and under	252	41.9
Junior high school and higher	350	58.1
**Household income**		
Poor	77	12.8
Wealthy	525	87.2
**Health insurance ownership**		
None	2	0.3
Compulsory health insurance	124	20.6
Government budget subsidy	327	54.3
Voluntary health insurance	149	24.8
**First point of care**		
Commune health centers	561	93.2
District health centers	41	6.8
**Health-related quality of life**		
Low	348	57.8
High	254	42.2
**Diagnosis of NCDs**		
Hypertension only	352	58.5
Diabetes mellitus only	115	19.1
Both hypertension and diabetes	135	22.4
**Charlson co-morbidity index**		
Mild	534	88.7
Moderate	56	9.3
Severe	12	2.0
**Duration of disease**		
≤ 5	386	67.0
6–10	120	20.8
> 10	70	12.2
**Disease control**		
Well control	186	30.9
Poor control	415	69.1

^a^Remote areas include rural and mountainous regions

Tables 2 and 3 show the description of COC measured by COCI and NCQ scores among the study population. COCI had an overall mean value of 0.77 (SD = 0.25), with 52.7% achieving a high COCI score (> 0.75), while the mean NCQ score was 3.59 (SD = 0.7) with 31.2% achieving a high NCQ score (> 4.0). There was no statistically significant difference in COCI by gender, while males reported higher NCQ scores than females (*p* < 0.05). People under 45 years old had the lowest COCI and NCQ scores. COCI score was lower (*p* < 0.001), and the NCQ was higher (*p* < 0.01) in remote areas compared to urban areas. There was a slight decrease in NCQ scores from low to high health-related quality of life (*p* < 0.05). While both COCI and NCQ scores showed slight increases with adherence to healthy behaviors, only smoking behavior had a significant association with COCI (p < 0.05). People under the voluntary health insurance scheme had the highest COCI (*p* < 0.001) and the lowest NCQ score (p < 0.001) among the study population.

**Table 2:** Distribution of COCI and NCQ scores by respondents’ characteristics

Characteristics (*n* = 602)	COCI				NCQ score			
	Mean score Mean (SD)	Low n (%)	High n (%)	*p*^a^	Mean score Mean (SD)	Low n (%)	High n (%)	*p*^a^
**Sample size**		285 (47.3)	317 (52.7)	–		414 (68.8)	188 (31.2)	–
**Average score (Mean (SD))**	0.77 (0.25)	0.55 (0.18)	0.97 (0.07)	–	3.59 (0.7)	3.27 (0.6)	4.29 (0.26)	–
**Gender**								
Male	0.79 (0.23)	120 (45.6)	143 (54.4)	*0.255*	3.64 (0.68)	171 (65.0)	92 (35.0)	* 0.049*
Female	0.75 (0.26)	165 (48.7)	174 (51.3)		3.55 (0.72)	243 (71.7)	96 (28.3)	
**Age**								
< 45	0.66 (0.34)	18 (56.3)	14 (43.8)	* 0.349*	3.43 (0.78)	23 (71.9)	9 (28.1)	*0.213*
45–64	0.76 (0.24)	129 (50.0)	129 (50.0)		3.61 (0.73)	169 (65.5)	89 (34.5)	
65–74	0.79 (0.24)	74 (42.8)	99 (57.2)		3.55 (0.74)	117 (67.6)	56 (32.4)	
≥ 75	0.78 (0.24)	64 (46.0)	75 (54.0)		3.63 (0.57)	105 (75.5)	34 (24.5)	
**Area**								
Urban	0.82 (0.22)	62 (35.6)	112 (64.4)	< 0.001	3.47 (0.68)	135 (77.6)	39 (22.4)	*0.002*
Remote	0.74 (0.26)	223 (52.1)	205 (47.9)		3.64 (0.71)	279 (65.2)	149 (34.8)	
**Highest education**								
Primary education and under	0.76 (0.25)	122 (48.4)	130 (51.6)	*0.358*	3.53 (0.75)	179 (71.0)	73 (29.0)	*0.177*
Junior school and above	0.77 (0.25)	163 (46.6)	187 (53.4)		3.63 (0.66)	235 (67.1)	115 (32.9)	
**Household income**								
Poor	3.47 (0.74)	32 (41.6)	45 (58.4)	*0.167*	0.79 (0.24)	54 (70.1)	23 (29.9)	*0.448*
Wealthy	3.61 (0.69)	253 (48.2)	272 (51.8)		0.76 (0.25)	360 (68.6)	165 (31.4)	
**Health insurance ownership**								
None	0.68 (0.18)	1 (50.0)	1 (50.0)	< 0.001	4.52 (0.03)	0 (0.0)	2 (100.0)	< 0.001
Compulsory	0.71 (0.18)	92 (74.2)	32 (25.8)		3.94 (0.57)	61 (49.2)	63 (50.8)	
Government budget subsidy	0.78 (0.25)	140 (42.8)	187 (57.2)		3.55 (0.72)	228 (69.7)	99 (30.3)	
Voluntary	0.79 (0.28)	52 (34.9)	97 (65.1)		3.38 (0.64)	125 (83.9)	24 (16.1)	
**QoL**								
Low (< 0.91)	0.76 (0.25)	169 (48.6)	179 (51.4)	*0.268*	3.65 (0.71)	226 (64.9)	122 (35.1)	*0.011*
High (≥ 0.91)	0.78 (0.24)	116 (45.7)	138 (54.3)		3.51 (0.69)	188 (74.0)	66 (26.0)	
**Alcohol assumption**								
Yes	0.79 (0.25)	54 (45.4)	65 (54.6)	*0.354*	3.63 (0.7)	75 (63.0)	44 (37.0)	*0.082*
No	0.76 (0.25)	231 (47.8)	252 (52.2)		3.58 (0.7)	339 (70.2)	144 (29.8)	
**Active smoking**								
Yes	0.78 (0.25)	86 (42.2)	118 (57.8)	*0.041*	3.6 (0.71)	136 (66.7)	68 (33.3)	*0.24*
No	0.76 (0.25)	199 (50.0)	199 (50.0)		3.6 (0.7)	278 (69.8)	120 (30.2)	
**Physical activities**								
Yes	0.77 (0.25)	214 (46.6)	245 (53.4)	*0.295*	3.58 (0.71)	311 (67.8)	148 (32.2)	*0.196*
No	0.76 (0.24)	71 (49.7)	72 (50.3)		3.61 (0.67)	103 (72.0)	40 (28.0)	

*p*^a^:* p*-value of Chi-square test results in analyzing the association between groups of high and low COC and demographic characteristics

**Table 3:** Continuity of care by clinical characteristics among study population

Characteristics (*n* = 602)	COCI				NCQ score			
	Mean score Mean (SD)	Low n (%)	High n (%)	*p*^a^	Mean score Mean (SD)	Low n (%)	High n (%)	*p*^a^
**BMI**								
Underweight	0.77 (0.27)	26 (45.6)	31 (54.4)	*0.96*	3.36 (0.76)	46 (80.7)	11 (19.3)	*0.074*
Normal weight	0.77 (0.24)	200 (47.6)	220 (52.4)		3.64 (0.67)	279 (66.4)	141 (33.6)	
Overweight/ Obesity	0.76 (0.27	59 (47.2)	66 (52.8)		3.59 (0.7)	89 (71.2)	36 (28.8)	
**Waist circumference**								
Normal	0.82 (0.24)	251 (51.0)	241 (49.0)	*< 0.001*	3.43 (0.7)	332 (67.5)	160 (32.5)	*0.09*
At risk	0.76 (0.25)	34 (30.9)	76 (69.1)		3.62 (0.7)	82 (74.5)	28 (25.5)	
**Blood pressure**								
Normal	0.75 (0.27)	104 (48.6)	110 (51.4)	*0.135*	3.58 (0.73)	141 (65.9)	73 (34.1)	*0.508*
Elevated blood pressure	0.8 (0.25)	27 (36.5)	47 (63.5)		3.55 (0.63)	53 (71.6)	21 (28.4)	
High blood pressure	0.77 (0.24)	154 (49.0)	160 (51.0)		3.61 (0.7)	220 (70.1)	94 (29.9)	
**Blood glucose**								
Normal	0.78 (0.28)	56 (36.1)	99 (63.9)	*0.002*	3.5 (0.68)	114 (73.5)	41 (26.5)	*0.233*
Impaired glucose tolerance	0.75 (0.23)	106 (55.2)	86 (44.8)		3.67 (0.7)	125 (65.1)	67 (34.9)	
High blood glucose	0.77 (0.25)	121 (48.8)	127 (51.2)		3.59 (0.71)	168 (67.7)	80 (32.3)	
**Diagnosis of NCDs**								
Hypertension only	0.78 (0.26)	156 (44.3)	196 (55.7)	*0.032*	3.6 (0.67)	244 (69.3)	108 (30.7)	*0.039*
Diabetes only	0.73 (0.24)	67 (58.3)	48 (41.7)		3.67 (0.78)	69 (60.0)	46 (40.0)	
Both hypertension and diabetes	0.77 (0.22)	62 (45.9)	73 (54.1)		3.51 (0.69)	101 (74.8)	34 (25.2)	
**Duration of disease**								
≤ 5	0.76 (0.25)	189 (49.0)	197 (51.0)	*0.028*	3.6 (0.71)	257 (66.6)	129 (33.4)	*0.316*
6–10	0.78 (0.2)	62 (51.7)	58 (48.3)		3.57 (0.68)	86 (71.7)	34 (28.3)	
> 10	0.81 (0.25)	23 (32.9)	47 (67.1)		3.49 (0.74)	52 (74.3)	18 (25.7)	
**Number of pills per day**								
None	0.62 (0.41)	25 (51.0)	24 (49.0)	*0.467*	3.44 (0.73)	40 (81.6)	9 (18.4)	*0.036*
1–2	0.78 (0.22)	213 (48.3)	228 (51.7)		3.64 (0.69)	290 (65.8)	151 (34.2)	
≥ 3	0.78 (0.23)	43 (42.2)	59 (57.8)		3.5 (0.73)	75 (73.5)	27 (26.5)	
**Charlson co-morbidity index**								
Mild	0.77 (0.25)	253 (47.4)	281 (52.6)	*0.576*	3.61 (0.69)	361 (67.6)	173 (32.4)	*0.118*
Moderate	0.75 (0.25)	28 (50.0)	28 (50.0)		3.51 (0.68)	42 (75.0)	14 (25.0)	
Severe	0.82 (0.25)	4 (33.3)	8 (66.7)		3.13 (1.16)	11 (91.7)	1 (8.3)	
**Disease control**								
Well control	0.81 (0.27)	59 (31.7)	127 (68.3)	*< 0.001*	3.48 (0.66)	138 (74.2)	48 (25.8)	* 0.032*
Poor control	0.75 (0.24)	225 (54.2)	190 (45.8)		3.64 (0.71)	275 (66.3)	140 (33.7)	
**Usual health facility**								
Commune health centers	0.77 (0.25)	223 (48.3)	239 (51.7)	*0.71*	3.68 (0.67)	298 (64.5)	164 (35.5)	*< 0.001*
District health centers	0.76 (0.26)	50 (44.2)	63 (55.8)		3.3 (0.71)	96 (85.0)	17 (15.0)	
Secondary/Tertiary care	0.76 (0.24)	12 (44.4)	15 (55.6)		3.23 (0.79)	20 (74.1)	7 (25.9)	
**Number of emergency visits**								
None	0.77 (0.25)	261 (47.1)	293 (52.9)	*0.634*	3.59 (0.7)	383 (69.1)	171 (30.9)	*0.803*
1–2 times	0.77 (0.23)	20 (47.6)	22 (52.4)		3.6 (0.77)	27 (64.3)	15 (35.7)	
≥ 3 times	0.65 (0.24)	4 (66.7)	2 (33.3)		3.49 (0.7)	4 (66.7)	2 (33.3)	
**Number of hospital admission**								
None	0.77 (0.25)	236 (46.5)	271 (53.5)	*0.356*	3.6 (0.71)	345 (68.0)	162 (32.0)	*0.469*
1–2 times	0.74 (0.24)	41 (49.4)	42 (50.6)		3.57 (0.69)	59 (71.1)	24 (28.9)	
≥ 3 times	0.66 (0.25)	8 (66.7)	4 (33.3)		3.45 (0.5)	10 (83.3)	2 (16.7)	

*p*^a^:* p*-value of Chi-square test results in analyzing the association between groups of high and low COC and clinical characteristics.

Table 3 showed no statistically significant difference in COCI and NCQ scores among people with different blood pressure levels, while a slight decrease in COCI was observed from normal to high blood glucose levels (*p* < 0.01). In the last 12 months, the average number of medical encounters was 14.8 (SD = 10.4), and people had low rates of emergency department encounters or hospital admissions. Among participants, people with only diabetes had the lowest COCI but the highest score of NCQ (*p* < 0.05). Both COCI and NCQ increased gradually by increasing the number of daily pills. In contrast to NCQ, the better control of disease people achieved, the higher score of COCI they had (*p* < 0.001).

The paired sample t-test was used to compare the NCQ score perceived by participants between general practitioners and specialists. Findings showed that general practitioners offered higher informational COC than specialists (p < 0.01), and the level of team/cross-boundary continuity was higher within the primary care team compared to between primary and specialist care (p < 0.001). We also compared the COC perceived by those with diabetes, hypertension, and both diseases within each subscale of the NCQ (Table 4). People with only T2DM had statistically significantly higher scores in Personal continuity - Specialist knows me, Personal continuity - Specialist shows commitment and Team/Cross-boundary within the specialist care subscales compared to those with hypertension and both diseases.

**Table 4:** Distribution of COC across care levels measured for people with hypertension, diabetes, and both diseases

Subscales of NCQ	Overall		Diabetes		Hypertension		Hypertension and Diabetes	
	*n*	*Mean (SD)*	*n*	*Mean (SD)*	*n*	*Mean (SD)*	*n*	*Mean (SD)*
**Personal continuity/ Informational continuity**								
GP knows me	602	3.45 (0.75)	115	3.38 (0.76)	352	3.48 (0.76)	135	3.44 (0.74)
GP shows commitment	602	3.1 (0.8)	115	3.16 (0.8)	352	3.11 (0.79)	135	3.04 (0.8)
Specialist knows me	295	3.15 (0.79)	63	3.42 (0.54)	169	3.07 (0.83)**	63	3.09 (0.81)*
Specialist shows commitment	295	3.02 (0.86)	63	3.37 (0.7)	169	2.92 (0.89)***	63	2.96 (0.82)**
**Team/Cross-boundary continuity**								
Within primary care	602	3.5 (0.73)	115	3.48 (0.68)	352	3.52 (0.74)	135	3.47 (0.74)
Within specialist care	295	3.46 (0.84)	63	3.72 (0.7)	169	3.39 (0.89)**	63	3.38 (0.78)*
Within primary and specialist care	295	3.08 (0.89)	63	3.25 (0.81)	169	3.07 (0.92)	63	2.97 (0.89)

*GP *General Practitioners/Primary care providers

ANOVA tests were performed for each subscale of NCQ. Respondents with diabetes were used as the reference group. Statistically significant results were bolded with *p < 0.05, **p < 0.01, ***p < 0.001

Table 5 summarizes the results of the multivariable logistic regression model for the proportion of people who achieved a high level of COCI and NCQ. Participants in the urban area (OR: 1.75, CI: 1.17–2.63), having high blood pressure (OR: 3.83, CI: 2.2–6.8) and living with chronic diseases for more than 10 years had increased odds of having a higher COCI level. Moreover, COCI was consistently related to reduced odds of hospital admission, poor disease control, and impaired glucose tolerance. In terms of NCQ, people who reported higher scores on NCQ had no prior visit to the emergency department (OR: 3.75, CI: 1.25–10.22), more than 10 times of health encounters during the last 12 months (OR: 4.4, CI: 2.08–9.3), and poor control of disease (OR: 2.59, CI: 1.31–5.12).

**Table 5:** Factors related to the high level of continuity of care

Variables	COCI score			NCQ score		
	B	OR (95%, CI)	*p*-value	B	OR (95%, CI)	*p*-value
**Gender**						
Male		1	0.663		1	0.032
Female	−0.08	0.92 (0.64–1.33)		−0.43	0.65 (0.44–0.96)	
**Area**						
Remote		1	0.007		1	0.005
Urban	0.56	1.75 (1.17–2.63)		−0.65	0.52(0.33–0.83)	
**Blood glucose level**						
Normal		**1**			**1**	
Impaired glucose tolerance	−0.69	0.5 (0.31–0.82)	0.006	0.4	1.49 (0.87–2.56)	0.145
High blood glucose	0.2	1.22 (0.72–2.08)	0.462	−0.11	0.9 (0.5–1.63)	0.725
**Blood pressure level**						
Normal		1	< 0.001		1	0.001
High blood pressure	1.34	3.83 (2.2–6.8)		−1.0	0.37 (0.2–0.67)	
**Usual healthcare facility**						
Commune health centers		1			1	
District health centers	0.14	1.16 (0.72–1.85)	0.549	−1.11	0.35 (0.18–0.61)	< 0.001
Secondary/Tertiary care	0.22	1.25 (0.52–3.0)	0.623	−0.45	0.64 (0.25–1.66)	0.358
**Number of hospitalization**						
None		1			1	
1–2 times	−0.24	0.79 (0.4–1.54)	0.786	−0.74	0.48 (0.2–1.12)	0.09
≥ 3 times	−1.66	0.2 (0.04–0.93)	0.04	−1.58	0.21 (0.03–1.25)	0.086
**Emergency encounter**						
Yes		1	0.79		1	0.018
No	−0.12	0.89 (0.36–2.18)		1.27	3.57 (1.25–10.22)	
**Number of total medical encounters**						
* ≤ 5*		1			1	
6–10	−0.39	0.67 (0.31–1.45)	0.313	0.66	1.94 (0.71–5.3)	0.194
> 10	0.11	1.12 (0.65–1.91)	0.687	1.48	4.4 (2.08–9.3)	< 0.001
**Duration of disease**						
≤ 5		1			1	
6–10	−0.12	0.88 (0.56–1.39)	0.594	−0.25	0.78 (0.47–1.28)	0.32
> 10	0.72	2.06 (1.15–3.67)	0.015	− 0.44	0.65 (0.35–1.21)	0.172
**Charlson co-morbidity index**						
Mild		1	0.102		1	0.074
Moderate, Severe	1.08	2.95 (0.81–10.8)		−1.98	0.14 (0.02–1.21)	
**Disease control**						
Well control		1	< 0.001		1	0.006
Poor control	−1.98	0.14 (0.07–0.27)		0.95	2.59 (1.31–5.12)	
**Health-related quality of life**						
Low		1	0.733		1	0.054
High	−0.07	0.94 (0.64–1.37)		− 0.41	0.67 (0.44–1.01)	

Variables with *P*-value < 0.2 in the bi-variable analysis were selected for multivariable analysis

Table 6 presents the pair-wise correlation of COC measurements. Overall, the COCI was weakly correlated with NCQ and the *Personal continuity - Primary care provider knows me* subscale (p < 0.01). It also showed that the correlation coefficients between the NCQ subscales and the total NCQ score were high, ranging from 0.68–0.79 (*p* < 0.01). Other positive correlations were found between different subscales of NCQ (p < 0.01), except for correlations between the *Personal continuity- Primary care provider knows me* subscale and other subscales regarding specialist care.

**Table 6:** Pearson correlation coefficients between Continuity of care Index and domains of Nijmegen continuity of care

	COCI	NCQ	Subscale 1_GP	Subscale 2_GP	Subscale 3_GP	Subscale 1_SP	Subscale 2_SP	Subscale 3_SP	Subscale 3_GP&SP
COCI	1								
NCQ	−0.12^**^	1							
Subscale 1_GP	0.09^**^	0.68^**^	1						
Subscale 2_GP	−0.06	0.74^**^	0.56^**^	1					
Subscale 3_GP	0.04	0.71^**^	0.51**	0.56^**^	1				
Subscale 1_SP	−0.05	0.77^**^	0.06	0.35**	0.22^**^	1			
Subscale 2_SP	−0.05	0.72^**^	0.02	0.34**	0.19**	0.74^**^	1		
Subscale 3_SP	−0.01	0.75^**^	0.11	0.36**	0.38^**^	0.6^**^	0.54**	1	
Subscale 3_GP&SP	0.001	0.79^**^	0.23**	0.36^**^	0.34^**^	0.57^**^	0.60^**^	0.54^**^	1

Subscale 1_GP: Personal continuity- Primary care provider knows me; Subscale 2_GP: Personal continuity- Primary care provider shows commitment; Subscale 3_GP: Team/Cross-boundary continuity Within primary care; Subscale 1_SP: Personal continuity- Specialist knows me; Subscale 2_SP: Personal continuity- Specialist shows commitment; Subscale 3_SP: Team/Cross-boundary continuity Within specialist care; Subscale 3_GP&SP: Team/Cross-boundary continuity Within primary and specialist care. *p < 0.05, **p < 0.01

## Discussion

COC plays a crucial role in ensuring the quality, safety, and efficiency of chronic care. Our study indicated that the COC was not sufficiently achieved by most people with diabetes and hypertension, as documented in the existing literature. Using NCQ to measure COC across care levels, similar to our results, a study in the Netherlands found a mean COC value for their population of 3.38 (SD = 0.72) [41]. The COCI results in our study were consistent with a previous study in Korea (2013) (COCI for four-year follow-up in T2DM: 0.75) [42], lower than another study in Korea (2019) (COCI among diabetic people: 0.83) [21], and higher than a study in Italy (2016) (COCI for multiple chronic conditions: 0.44) [43], in China (2017–2019) (COCI for hypertension and T2DM: 0.58) [20] and in Norway (2021) (COCI for T2DM: 0.67 and COCI for heart failure: 0.77) [44]. Compared to studies using the same cutoff point of COCI, our study had a lower proportion of high COCI than others [29, 37]. Differences in sample size and health conditions of the study population may explain the variations in COC results. Previous studies predominantly utilized claims data or national health insurance databases with a high proportion of people with co-morbidity requiring long-term and continuous care, unlike our study population with a low proportion of co-morbidity. Additionally, these studies were conducted in hospital settings, while our research focused on primary care. The insufficient COC observed in our study and previous studies raises concerns about fragmented care for people with chronic diseases. It emphasizes the need for increased efforts to promote continuity in chronic care and implement integrated care programs in primary care.

An interesting result of our study is that remote areas exhibited lower levels of COCI and higher NCQ scores compared to urban areas. The lower COCI is consistent with the lack of health workforces and poor accessibility to health care providers, acknowledged in available evidence [45, 46]. Otherwise, considering patients’ perceived COC, people in remote areas could have closer relationships with their primary care providers, resulting in higher perceived informational and management continuity. In contrast, in urban areas, people may have more options for healthcare providers and facilities, potentially resulting in fragmented care. The discrepancies observed in COC results between the Bice - Boxerman continuity of care index and the Nijmegen continuity of care questionnaire might be due to the extent of the COC concept measured in our study. Apparently, COC should be seen within the context of healthcare service organization and delivery systems. Thus, simply analyzing sequential visits to the same provider is insufficient; it is crucial to additionally assess patients’ perspectives on the healthcare process they received. Our study employed both instruments to capture various aspects of COC; COCI provided better insight into healthcare utilization fragmentation among individuals with NCDs, while NCQ highlighted how well patients received COC across primary care and hospital care. While this issue needs further investigation, our study approach can be applicable to other countries and settings with similar healthcare delivery systems.

Relevant to our study, Hopstaken JS et al. [41], Hetlevik Ø et al. [44], and Arnold C [47] also described the higher COC of primary care compared to hospital/specialist care. The advantage of a broad network and the critical role of primary care in providing essential and longitudinal care for hypertension, diabetes, and other NCDs are indisputable. The widespread availability of CHCs throughout Vietnam facilitates easier access to primary care services, fostering stronger patient-provider relationships and mutual trust. In contrast, a broader range of services provided by DHCs or upper-level facilities may lead to patients receiving care from multiple providers, resulting in disjointed care and weak continuity. A study across three provinces in Central Vietnam with 1662 residents found that CHCs provided better ongoing and coordinated care, although with lower accessibility and readability of services compared to higher-level public and private health facilities [48]. Therefore, strengthening robust primary care through improving service availability and readiness for chronic care and increasing the number of well-trained primary care providers, particularly in rural areas, have been recommended as top-priority solutions in our own and other studies [7, 45, 48]. We also propose that the health authority and care providers should implement telemedicine and virtual chronic care services with a financially supported mechanism to enhance equitable access to health services and boost the COC for people with chronic diseases.

Another concept of COC for people with complex care needs is management continuity which relates to the interconnectedness of care providers along the chronic care pathway. Our results align with previous studies in which patients reported limited continuity between primary and specialist care [[24], 41, 47]. De Witt A et al. conducted a study in Australia that also emphasized insufficient partnership, communication and timely information exchange between primary and hospital cancer services from the health professionals’ perspectives [49]. In Thua Thien province, a qualitative study showed a lack of perception and practice toward interprofessional collaboration in chronic care among primary care professionals, hindering a shared decision-making approach in people-centered care [50]. To foster holistic, people-centered care and enhance continuity, we strongly advocate for implementing team-based and multidisciplinary care and comprehensive training programs in interprofessional collaboration toward chronic diseases. Upgrading electronic medical record and personal health record systems and installing an interactive referral system for primary-specialist care coordination are other promising interventions to improve care coordination between providers and ensure patients receive appropriate follow-up care.

In this study, we highlighted a high proportion of poor control of T2DM and hypertension, approximately two-thirds of the study population. Our findings support existing literature, indicating an association between COC and disease control. Whereas higher COCI was found among people with better disease control [21, 51], higher NCQ was observed among people with poor management [24, 47]. This suggests that better disease control may be linked to improved self-efficacy and seeking care from specific providers when needed, resulting in higher COCI levels and lower disjointed care. Otherwise, people with poor disease control often require more attention and coordination between primary and specialist care. Consequently, they could have more visits for follow-up care and shorter periods between visits to the healthcare provider compared to those with better disease control. These aspects could contribute to higher NCQ scores and better perceived COC.

Our findings align with the literature, illustrating that high COC was associated with reduced hospital and emergency department admissions [19, 21, 24] and better-controlled blood pressure and blood glucose levels [21, 51]. COC has been acknowledged to facilitate higher patient self-care behaviors and adherence to physicians’ recommendations and treatment regimes, which could improve disease control and reduce preventable hospital hospitalizations and complications [19, 52]. Studies by Ludt et al. [53] and Arnold et al. [47] indicated that people receiving lifestyle counselling and involved in the shared decision-making process had higher odds of better COC. Additional services such as behavior change counselling, self-care consultation, and patient empowerment could strengthen patient-provider relationship, enhance treatment adherence, and improve care provider commitment to patient health conditions.

The findings of this study hold significant relevance for advocating policies in the reform of healthcare systems for NCDs, not only in Vietnam but also in other countries that encounter similar circumstances. Our study used a precise approach that examined the multi-dimensions of the COC concept in primary care settings, however, we did not include an analytical hierarchy to weight and aggregate the composite COC indicators/subscales. Moreover, our study has a limitation as a cross-sectional descriptive study based on self-reported questionnaires. While we examined various T2DM and hypertension-related outcomes to identify predictors of higher COC, the possibility of recall bias restricts our ability to evaluate a cause-effect relationship between COC and health outcomes and health services utilization. Further studies using medical records or national health insurance data could derive better insight into the association of COC with health service utilization and the cost-effectiveness of chronic care.

## Conclusions

The continuity of care perceived by people with hypertension and/or type 2 diabetes mellitus is insufficient. Primary care demonstrates higher continuity of care than higher levels of care, with limited continuity between primary and specialist care. Our results demonstrate associations between continuity of care and hospital admissions, emergency department visits, and disease control. To enhance chronic care in primary care settings, we recommend prioritizing the quality and accessibility of NCDs services in primary care, reducing the geographical gaps in service delivery, improving collaboration between healthcare providers through a multidisciplinary team-based and integrated care approach, and implementing patient education and shared-decision making interventions. Further investigations should be carried out with a longitudinal healthcare database to observe more concrete indicators of COC.

### Supplementary Information

**Additional file 1.** Geographic Map of Selected Communes and Districts in the Study.

## Data Availability

The data presented in this study are available from the corresponding authors upon reasonable request.

## Abbreviations

BP: Blood pressure
CCI: Charlson co-morbidity index
CHC: Commune health center
COC: Continuity of care
COCI: Bice - Boxerman continuity of care index
DHC: District-level health facility
EQ-5D-5L: EuroQOL-5 Dimensions-5 Levels
GP: General practitioner
QoL: Health-related quality of life
LMICs: Low and middle-income countries
NCDs: Non-communicable diseases
NCQ: Nijmegen continuity of care questionnaire
OR: Odds ratio
T2DM: Type 2 diabetes mellitus
WHO: World Health Organization
