# Supplementary figures and images for: Continuity of primary care for type 2 diabetes and hypertension and its association with health outcomes and disease control: insights from Central Vietnam
Source: BMC Public Health. 2024 Jan 2;24:34. doi: 10.1186/s12889-023-17522-6 (PMC10763071; doi:10.1186/s12889-023-17522-6)

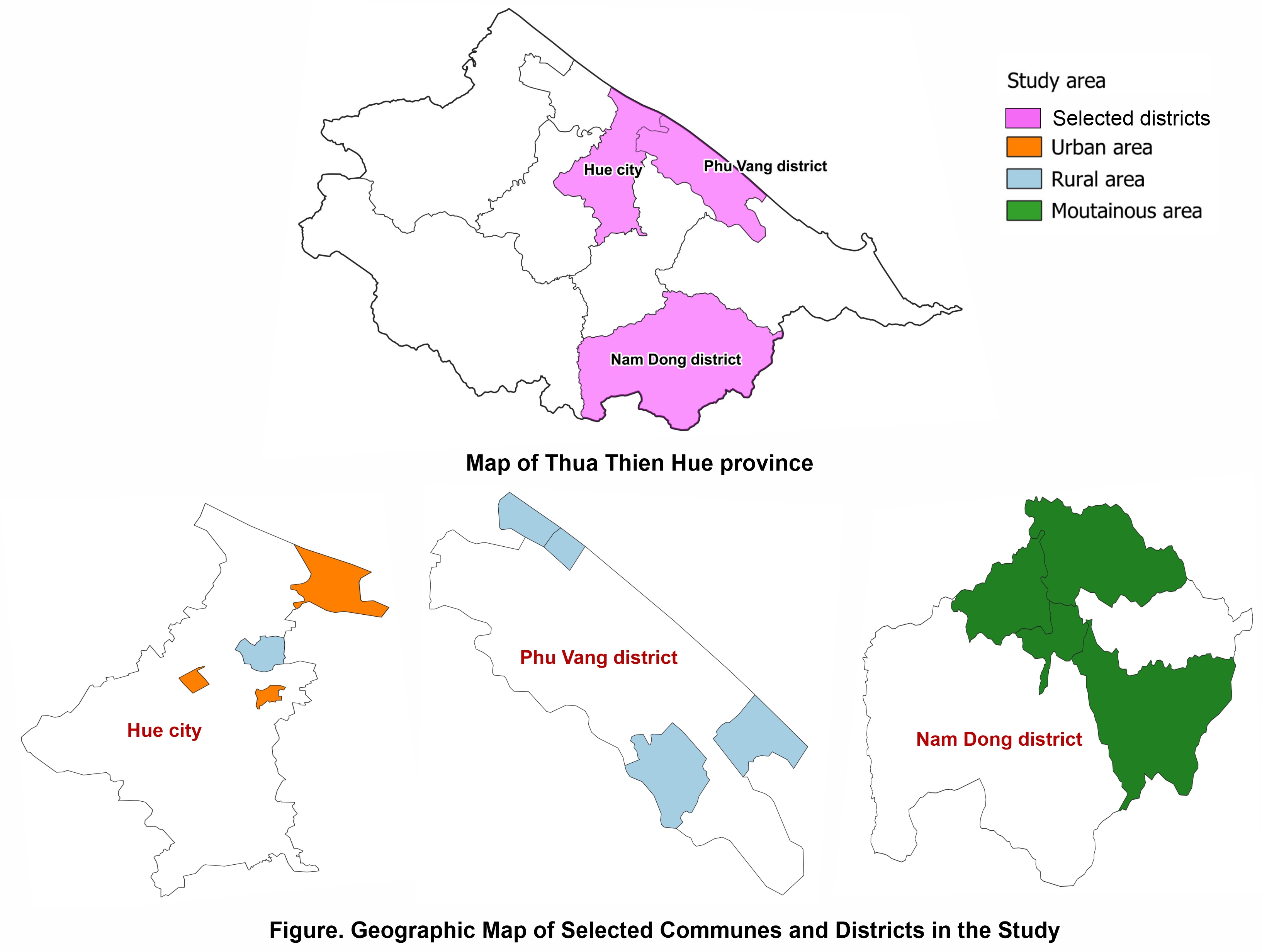

Supplement: Supplementary file 1 — Additional file 1. Geographic Map of Selected Communes and Districts in the Study. [file 12889_2023_17522_MOESM1_ESM.jpg]
